# Alternative Complement Pathway Deregulation Is Correlated with Dengue Severity

**DOI:** 10.1371/journal.pone.0006782

**Published:** 2009-08-26

**Authors:** Eduardo J. M. Nascimento, Ana M. Silva, Marli T. Cordeiro, Carlos A. Brito, Laura H. V. G. Gil, Ulisses Braga-Neto, Ernesto T. A. Marques

**Affiliations:** 1 Division of Infectious Diseases, Department of Medicine, The Johns Hopkins School of Medicine, Baltimore, Maryland, United States of America; 2 Virology and Experimental Therapy Laboratory, Aggeu Magalhães Research Center-CPqAM/FIOCRUZ, Recife, Pernambuco, Brazil; 3 Department of Electrical and Computer Engineering, Texas A&M University, College Station, Texas, United States of America; 4 Department of Pharmacology and Molecular Sciences, The Johns Hopkins School of Medicine, Baltimore, Maryland, United States of America; BMSI-A*STAR, Singapore

## Abstract

**Background:**

The complement system, a key component that links the innate and adaptive immune responses, has three pathways: the classical, lectin, and alternative pathways. In the present study, we have analyzed the levels of various complement components in blood samples from dengue fever (DF) and dengue hemorrhagic fever (DHF) patients and found that the level of complement activation is associated with disease severity.

**Methods and Results:**

Patients with DHF had lower levels of complement factor 3 (C3; *p* = 0.002) and increased levels of C3a, C4a and C5a (*p*<0.0001) when compared to those with the less severe form, DF. There were no significant differences between DF and DHF patients in the levels of C1q, immunocomplexes (CIC-CIq) and CRP. However, small but statistically significant differences were detected in the levels of MBL. In contrast, the levels of two regulatory proteins of the alternative pathway varied widely between DF and DHF patients: DHF patients had higher levels of factor D (*p* = 0.01), which cleaves factor B to yield the active (C3bBb) C3 convertase, and lower levels of factor H (*p* = 0.03), which inactivates the (C3bBb) C3 convertase, than did DF patients. When we considered the levels of factors D and H together as an indicator of (C3bBb) C3 convertase regulation, we found that the plasma levels of these regulatory proteins in DHF patients favored the formation of the (C3bBb) C3 convertase, whereas its formation was inhibited in DF patients (*p*<0.0001).

**Conclusion:**

The data suggest that an imbalance in the levels of regulatory factors D and H is associated with an abnormal regulation of complement activity in DHF patients.

## Introduction

The complement system plays both effector and regulatory roles in the innate and adaptative arms of the immune response. It is also a key factor in integrating these two defense systems. As part of the innate immune system, the complement system serves as the first line of defense against infectious agents; while as part of the adaptive immune system; it is involved in modulation of B- and T-cell function, maturation, and immunological memory. The complement system has been shown to have a complex role in *Flavivirus* infections, being considered either protective, by limiting viral replication, increasing the clearance of virus, and inhibiting antibody-dependent enhancement (ADE); or deleterious, by increasing disease severity when excessively activated, causing an exacerbated inflammatory response [1], [2]. The involvement of augmented complement activation in dengue pathophisiolgy was demonstrated by Bokisch et al. [3], however the specific mechanisms triggering the increased complement activation in dengue disease still need elucidation.

The complement system has three major pathways: the classical (CP), the alternative (AP), and the lectin (LP)-dependent pathways. The CP is often activated by antibody-antigen complexes [4] or by the C-reactive protein (CRP) [5], both of which interact with complement component C1q. In addition, the CP can be activated by phosphatidyl serine present in apoptotic bodies [6] in the absence of immune complexes. The LP is initiated by the mannose-binding lectins (MBL) that bind to complex carbohydrate residues on the surface of pathogens. The activation of the CP or the LP leads to activation of the (C4b2b) C3 convertase. The AP is activated by the binding of C3b, which is spontaneously generated by the hydrolysis of C3, on targeted surfaces. This surface-bound C3b is then recognized by factor B, forming the complex C3bB [7]. The C3bB complex, in turn, is cleaved by factor D to yield the active form of the C3 convertase of the AP (C3bBb). Both types of C3 convertases will cleave C3, forming C3b. C3b then either binds to more factor B, enhancing the complement activation through the AP (the so-called alternative or amplification loop), or leads to the formation of the active C5 convertase (C3bBbC3b or C4bC2bC3b), which cleaves C5 and triggers the late events that result in the formation of the membrane attack complex (C5b-9).

Dengue virus is a *Flavivirus* with four antigenically distinct serotypes (DENV-1 to DENV-4). Dengue virus infection is a global public health concern, with an estimated incidence of 50–100 million cases of dengue fever (DF) that result in 500,000 clinical cases of the life-threatening dengue hemorrhagic fever syndrome (DHF) and 24,000 deaths annually. DHF is characterized by vasculopathy, which results in a sudden plasma leakage that reduces the blood volume and may produce hypovolemic shock (dengue shock syndrome, DSS). The immunopathological mechanism responsible for the increased disease severity associated with DHF is only partially understood. Several lines of evidence have indicated that non-neutralizing anti-dengue antibodies play a modulatory role that involves ADE. However, other factors, including innate immunity, have also been shown to modulate the clinical outcome [8]–[10], and several studies have shown that complement factors are involved [11]. In a pioneering study, Bokisch et al. [3] measured the levels of nine complement factors (C1q, C1s, C2, C3, C4, C5, C6, C8 and factor B) in serum samples of DHF patients (grades I to IV). They found that levels of C3 were reduced in all DHF patients, and observed a correlation between C3 consumption and disease severity. In addition, the levels of C4 and factor B were reduced in the more severe DHF patients (grades III and IV). The authors observed a correlation between levels of C3 with the levels of factor B and C4 and based on these correlations the authors demonstrated the direct involvement of both the classical and the alternative pathways in dengue severity. However, neither the levels of C1q and C1s, nor the catabolic rate of C1q degradation presented a significant correlation with disease severity [3].

Other studies have shown that the dengue non-structural (NS)-1 protein can directly activate the complement cascade in the absence of antibody, and this activation is enhanced when anti-dengue antibodies are present [11]. In addition, clinical studies have shown that the levels of dengue NS1 and products of complement activation, including those with a known vascular effect, such as C3a, C5a, and sC5b-9, are present in higher levels in DHF patients before plasma leakage takes place, supporting the theory that complement activation is involved in the development of severe disease [12] and to deleterious patient outcomes [11], [13], [14]. Interestingly, the NS1 protein of West Nile virus, another flavivirus which does not normally induce vasculopathy, has been shown to bind to complement regulatory factor H [15] and use this mechanism as an immune evasion mechanism.

So far, it seems clear that the complement system is activated during dengue infection [11], [16]; however, it is not clear to what extent each of the three pathways (the CP, LP, and AP) contribute to the overall activation of the complement system, or what mechanisms are triggering the increased complement activation during dengue infection. In order to elucidate the contribution of the complement system to dengue clinical outcomes, we have quantified specific components of each of the three complement system pathways and compared their levels in dengue virus-infected patients with mild disease (DF) or severe disease (DHF), at different stages of infection (acute and convalescent), to those in normal volunteers. Our data show an important correlation between imbalances in the levels of factor H and factor D and increased dengue severity.

## Methods

### Patients

A cohort of suspected dengue cases admitted to three hospitals in the city of Recife in the state of Pernambuco, Brazil, has been established and described elsewhere [17]. Sequential blood samples collected during the first 30 days after the onset of symptoms were harvested and cryopreserved to supply serum and plasma samples at different stages of the infection. Plasma was collected in both heparin and citrate buffers and used according to the assay requirements. Dengue diagnosis was performed by combining virus isolation in C6/36 cells [18] and RT-PCR [19] with anti-dengue IgM/IgG ELISA. The classification of primary vs. secondary dengue infection was based on the kinetics of the IgM and IgG response, according to the following criteria: Primary infection was defined as the absence of specific anti-dengue IgG antibodies in the first serum samples during the acute phase, with IgM, virus, and/or viral RNA being detected and followed by the presence of anti-dengue IgG in convalescence serum samples. Secondary infection was defined as the detection of specific anti-dengue IgG in the first acute sample and an absence of anti-dengue IgM, associated with a positive RT-PCR and/or virus isolation [17], [20].

Dengue cases were clinically classified according to the World Health Organization (WHO) criteria into DF and DHF. DF cases were characterized by fever lasting up to 7 days and accompanied by at least two of the following symptoms: headache, retro-orbital pain, myalgia, arthralgia, and rash associated with a platelet level above 100,000/mm^3^. DHF cases were defined as having the same clinical manifestations as those in DF, but with evidence of hemorrhage, thrombocytopenia (platelet<100,000/mm^3^), and plasma leakage following deffervescence.

Since patient age, date of sample collection, and virus serotype and genotype are factors that can affect complement levels and potentially produce misleading results, we applied the following criteria in selecting patients: a) confirmed infection with the DENV-3 serotype (genotype III), b) age> = 13 years, and c) availability of a blood sample collected within 3 to 8 days of the onset of symptoms and defined as acute phase. A total of 74 dengue cases met the criteria: 47 patients with DF and 27 with DHF (grades I and II). In the case of the DF patients, 41% of the infections were primary and 59% secondary; in the case of the DHF patients, 58% were primary and 42% secondary (Table 1). A second set of samples from the same individuals collected 1 to 9 months after the onset of symptoms was used to represent physiologic levels after infection (termed “convalescent samples” in this report). Of the 74 cases identified, 48 (32 DF and 16 DHF patients) had convalescent samples available. The data presented in table 1 do not include all the information obtained from each patient and are not intended to define the clinical classification. The platelet data presented in the table give the count on the day of the sample used in this study and not the lowest count found in that particular patient. All DHF patients had thrombocytopenia at some time point. As a normal reference, we used samples from 30 healthy individuals, who were identified as eligible to receive yellow fever vaccine. The samples used here were collected from these individuals before immunization.

**Table 1:** Clinical and demographic profiles of patients infected with dengue virus (P) and of healthy controls (HC).

Patient ID	Age	Sex	Days of symptoms (acute phase)	Days of symptoms (conv. phase)	Type of Infection	Clinical Diagnosis	Platelet/mm^3^×10^3^ (acute phase)
P141	30	F	3	-	Primary	DF	-
P151	42	F	3	-	Primary	DF	209
P161	30	M	3	-	Primary	DF	-
P363	24	F	3	255	Primary	DF	246
P388	23	F	5	255	Primary	DF	172
P411	47	F	3	255	Primary	DF	160
P412	56	M	3	255	Primary	DF	206
P423	48	F	3	-	Primary	DF	153
P426	30	F	6	-	Primary	DF	224
P456	40	M	3	31	Primary	DF	214
P475	34	F	5	33	Primary	DF	178
P486	52	F	7	21	Primary	DF	350
P524	27	F	4	178	Primary	DF	149
P545	22	M	5	255	Primary	DF	162
P588	15	M	3	-	Primary	DF	148
P591	37	F	5	-	Primary	DF	224
P593	41	M	6	31	Primary	DF	277
P598	13	F	4	255	Primary	DF	-
P664	43	F	8	255	Primary	DF	257
P098	23	F	3	-	Secondary	DF	242
P107	41	M	5	-	Secondary	DF	141
P129	43	F	3	-	Secondary	DF	229
P301	13	F	4	24	Secondary	DF	215
P308	48	M	8	255	Secondary	DF	170
P310	33	F	3	31	Secondary	DF	90
P321	19	F	7	255	Secondary	DF	269
P330	19	F	4	255	Secondary	DF	143
P348	13	F	5	50	Secondary	DF	156
P356	62	M	8	31	Secondary	DF	232
P361	27	M	4	255	Secondary	DF	194
P374	18	M	8	32	Secondary	DF	196
P382	50	M	5	255	Secondary	DF	162
P435	40	M	5	32	Secondary	DF	153
P446	35	M	3	255	Secondary	DF	-
P481	49	F	5	255	Secondary	DF	140
P488	37	F	4	30	Secondary	DF	151
P504	46	F	3	-	Secondary	DF	200
P509	47	M	5	-	Secondary	DF	191
P511	53	F	8	-	Secondary	DF	223
P550	52	F	6	255	Secondary	DF	184
P578	35	M	5	-	Secondary	DF	232
P582	49	M	5	255	Secondary	DF	234
P600	26	M	6	-	Secondary	DF	284
P604	47	M	5	255	Secondary	DF	174
P620	58	M	8	255	Secondary	DF	141
P623	38	F	5	255	Secondary	DF	144
P659	32	F	8	255	Secondary	DF	274
P094	49	F	6	-	Primary	DHF	70
P102	25	F	5	255	Primary	DHF	72
P111	15	F	5	255	Primary	DHF	105
P128	45	F	3	255	Primary	DHF	148
P145	18	M	5	-	Primary	DHF	90
P165	33	F	4	-	Primary	DHF	178
P260	13	F	8	255	Primary	DHF	37
P305	45	M	4	255	Primary	DHF	80
P307	41	F	8	255	Primary	DHF	80
P339	47	F	6	-	Primary	DHF	87
P370	48	M	7	142	Primary	DHF	81
P430	34	F	6	-	Primary	DHF	49
P564	36	F	3	-	Primary	DHF	136
P613	47	F	5	255	Primary	DHF	42
P635	26	F	6	35	Primary	DHF	81
P104	47	F	8	255	Secondary	DHF	48
P125	19	F	6	-	Secondary	DHF	64
P193	22	F	5	-	Secondary	DHF	91
P206	12	M	3	255	Secondary	DHF	122
P277	48	M	3	255	Secondary	DHF	134
P420	76	F	4	-	Secondary	DHF	248
P428	57	M	3	255	Secondary	DHF	75
P543	27	F	7	-	Secondary	DHF	89
P549	58	F	6	32	Secondary	DHF	-
P586	56	F	5	255	Secondary	DHF	115
P629	17	M	4	19	Secondary	DHF	87
HC01	49	F	-	-	-	-	-
HC02	47	F	-	-	-	-	-
HC03	49	M	-	-	-	-	-
HC04	47	F	-	-	-	-	-
HC05	37	M	-	-	-	-	-
HC06	27	M	-	-	-	-	-
HC07	26	F	-	-	-	-	-
HC08	20	F	-	-	-	-	-
HC09	48	M	-	-	-	-	-
HC10	20	F	-	-	-	-	-
HC11	51	M	-	-	-	-	-
HC12	38	M	-	-	-	-	-
HC13	24	M	-	-	-	-	-
HC14	27	M	-	-	-	-	-
HC15	37	M	-	-	-	-	-
HC16	79	M	-	-	-	-	-
HC17	38	F	-	-	-	-	-
HC18	57	M	-	-	-	-	-
HC19	43	M	-	-	-	-	-
HC20	44	M	-	-	-	-	-
HC21	37	M	-	-	-	-	-
HC22	31	M	-	-	-	-	-
HC23	31	M	-	-	-	-	-
HC24	28	M	-	-	-	-	-
HC25	38	M	-	-	-	-	-
HC26	49	F	-	-	-	-	-
HC27	19	M	-	-	-	-	-
HC28	46	M	-	-	-	-	-
HC29	33	M	-	-	-	-	-
HC30	34	M	-	-	-	-	-

DF – dengue fever; DHF – dengue hemorrhagic fever; Prim – primary infection; Sec – secondary infection; SD – standard deviation. Note: the platelet count is the one obtained at the day of the sample used in the complement factor measurements. It is not the lowest level found in the patient.

### Ethical considerations

Written consent to participate in the study was obtained from each patient (or the patient's guardian) after a full explanation of the study was provided. All data were handled confidentially and anonymously. This study was reviewed and approved by the ethics committee of the Brazilian Ministry of Health (CONEP: 4909; Process n° 25000.119007/2002-03; CEP: 68/02). In addition, the Johns Hopkins University Institutional Review Board reviewed and approved the study as protocol JHM-IRB-3: 03-08-27-01.

### Quantitative ELISA Protocols

Purified human complement protein C1q (Quidel), a chicken polyclonal antiserum against human C1q (Abcam), and HRP-conjugated sheep anti-human C1q (AbD Serotec) were used to measure C1q in plasma samples. A standard curve was established using a nonlinear regression curve (R^2^>0.95) with seven standard concentrations; the range of detection was 0.0001–10.0 µg/mL. Purified human factor H (Calbiochem), a goat polyclonal antiserum against human factor H (Calbiochem), a mouse monoclonal antiserum [HYB 268] against human factor H (Abcam), and HRP-conjugated goat anti-mouse IgG (Jackson Immunoresearch) were used to measure factor H. A standard curve was established using a linear regression curve (R^2^>0.95) with five standard concentrations; the range of detection was 3–25 ng/mL. Purified human complement protein C3 (Quidel), a chicken polyclonal antiserum against human complement C3 (Genway), rabbit polyclonal antiserum against human C3 biotinylated (Abcam), and HRP-conjugated streptavidin (Rockland) were used to measure C3. A standard curve was established using a nonlinear regression curve (R^2^>0.95) with seven standard concentrations; the range of detection was 0.0001–10.0 µg/mL. The plasma samples were diluted so that the values obtained fell within the standard curves.

Nunc microplates were coated with primary antibody and diluted in carbonate/bicarbonate buffer, pH 9.6. After washing with PBS (pH 7.2) containing 0.05% Tween 20 (PBS-T), the plates were blocked with 5% BSA (Sigma) for 1 h at 37°C. They were then washed again and incubated for 2 h at 37°C with either the plasma samples from dengue patients or with purified protein, in duplicate. The plates were again washed and incubated for 1 h at 37°C with secondary antibody. When a tertiary step was necessary, the plates were washed and incubated for 1 h at 37°C with HRP-conjugated component (antibody or streptavidin). After a final wash, the plates were developed with TMB (BD Biosciences) and read at 450 nm in a microplate reader (Safari^2^, Tecan).

### Immunoassays for Complement Factor D, Anaphylotoxins, MBL Oligomers, Immunocomplexes, and C-Reactive Protein

Plasma levels of complement factor D, anaphylotoxins, MBL oligomers, and total IgG immunocomplexes (CIC-C1q) were measured using the Duo Set ELISA development kit for human factor D (R&D systems), the Cytometric Bead Array kit for human anaphylotoxin (BD Biosciences), the MBL oligomer ELISA kit (Innate Immunity Diagnostics), and the CIC-C1q ELISA kit (Quidel), respectively, according to manufacturer directions. C-reactive protein was measured by immunoturbimetry using a Hitachi 912 (Roche Diagnostics).

### Statistical Analysis

Statistical analysis and plotting were carried out using Prism version 4 and R version 2.9.0 for Macintosh. In order to ensure that the data are Gaussian-distributed, concentration values of each complement protein were log-transformed. Gaussianity of the log-tranformed data was confirmed by means of cumulative frequency distribution plots and the Kolmogorov-Smirnov test (data not shown). Two-tailed unpaired t-tests were then used to compare the averages of concentration of different proteins, whereas two-tailed paired t-tests were used to compare the levels of different complement components in the same individual at different stages of infection (acute and convalescence). Pearson's correlation was used to assess the co-variation among the groups analyzed. Differences were considered statistically significant when p<0.05.

In order to quantify the imbalance in levels of factor H and factor D across different diagnostic categories, log-ratios between the concentration of factor H over the concentration of factor D were computed. Factor D acts by cleaving BB in the C3bBB complex to yield active C3bBb, whereas factor H regulates C3bBb by displacing the Bb portion of this C3 convertase, thus having the opposing effect of inactivating the AP convertase. If the log-ratios in concentration of factor H over factor D tend to be larger in a given diagnostic category than in the healthy category, then we may conclude that the balance between factors D and H is less favorable for the formation of the C3bBb C3 convertase in that diagnostic category than in healthy individuals, whereas smaller log-ratios mean that the balance is more favorable. Thus, the log-ratios in concentration of factor H over factor D allow the study of how these two proteins are regulating the alternative pathway C3bBb C3 convertase levels, however it does not directly indicate the amount of active convertase.

## Results

### DHF patients show elevated complement activity and C3 consumption

**Figure 1:**
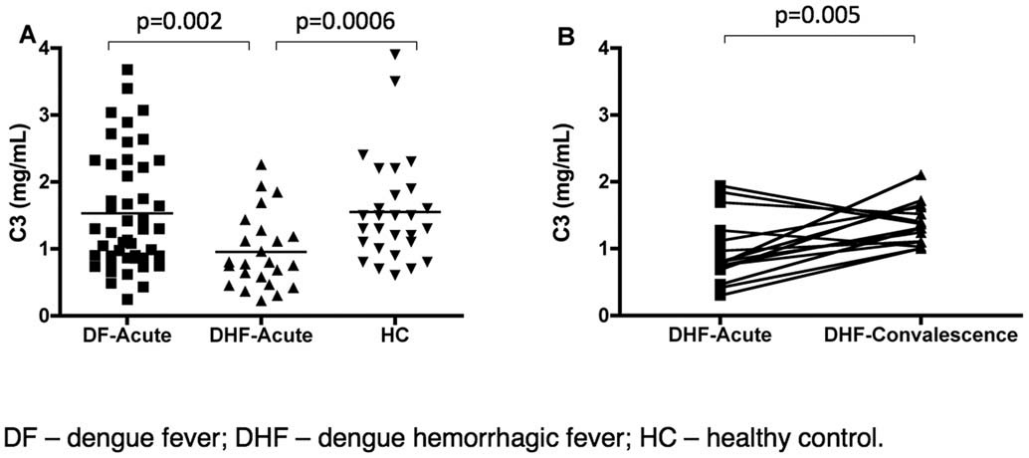
Levels of complement factor 3 (C3); plotted values are nonlog for ease of comprehension. (A) Plasma C3 levels in dengue-infected individuals during the acute phase (3–8 days after the onset of symptoms) and in healthy subjects. (B) Paired C3 levels of DHF patients during the acute and convalescent phases of dengue infection. The mean of C3 levels for acute and convalescence were 1.0+0.5 and 1.4+0.3 respectively. The C3 levels were measured by ELISA. Values shown are means and standard deviations. The p-values of two-tailed t-tests on the log-transformed data are indicated above each analysis.

During complement activation, C3 is cleaved to form C3a and C3b; when C3 is cleaved faster than it can be replaced, the levels of intact C3 decay. This phenomenon often occurs when the complement system is highly active. In the present study, we measured the levels of C3 in the DF and DHF patients during the acute phase and compared their levels to those of control patients (Figure 1A, Table 2). The C3 levels in the DF patients in the acute phase were nearly identical to those in the healthy subjects (1.5+/−0.9 mg/mL vs. 1.5+/−0.8 mg/mL, respectively). However, the C3 levels in DHF patients (1.0+/−0.6 mg/mL) were significantly (p = 0.002) lower than those in the DF patients during acute dengue infection and also significantly (p = 0.0006) lower than the C3 levels in the healthy control group. In order to determine whether the lower levels of C3 were indeed the result of high levels of complement system activity during infection, the C3 levels of the DHF patients were also measured during the convalescent phase (Figure 1B). The data showed a significant (p = 0.005) increase in the C3 levels of the DHF patients after they recovered from the acute infection, and these levels were not significantly different from the levels found in control patients, confirming that the C3 was consumed at higher levels in DHF patients than in DF patients during the acute stage of the dengue infection.

**Table 2:** Levels of different components of the complement system in plasma samples collected from dengue-infected patients with different clinical manifestations and at different stages of infection.

Complement proteins	Healthy Control	DF		DHF	
		Acute	Con	Acute	Con
C1q (µg/mL)	10±9	22±19*	11±7	18±23	9±3
MBL (µg/mL)	3.2±1.9	2.8±3.2	3.1±2.3	4.4±4.2	3.3±3.4
CRP (mg/dL)	0.2±0.2	1.1±1.6*	0.2±0.2	1.0±1.3*	0.1±0.2
CIC-C1q (µg Eq/mL)	ND	168±153	ND	149±115	ND
CFH (µg/mL)	206±123	233±192	202±103	142±92*	213±81
CFD (ng/mL)	761±204	625±343*	704±278	804±337	846±397
C3 (mg/mL)	1.5±0.8	1.5±0.9	1.3±0.3	1.0±0.6*	1.4±0.3

**Values shown are means and standard deviations.**

DF – dengue fever; DHF – dengue hemorrhagic fever; Acute – acute phase; Con – convalescent phase; CRP – C-reactive protein; CIC-C1q – IgG immunocomplex; CFD – factor D; CFH – factor H; HC – healthy control; ND – non-determined. * Indicate the significant (p≤0.05) differences between dengue patients with health controls.

In addition, we have performed analyses comparing the C3 levels on primary vs. secondary dengue infection and no significant differences were detected. Similarly we tested the effect of dengue infection history on the other complement factors we measured and we have not detected any influence within each clinical diagnosis assessed.

### DHF patients have elevated levels of the anaphylotoxins C3a and C5a

**Figure 2:**
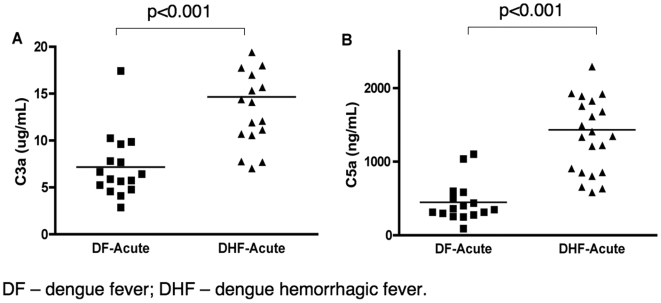
Levels of complement factors C3a and C5a; plotted values are nonlog for ease of comprehension. (A) Plasma levels of C3a and (B) C5a in dengue-infected individuals during the acute phase and in healthy subjects. C3a and C5a levels were measured by cytometric bead assay. Values shown are means and standard deviations. The p-values of two-tailed t-tests on the log-tranformed data are indicated above each analysis.

We also measured the plasma levels of C3a, a product of C3 cleavage, and C5a, a product of C5 cleavage, in samples from patients with confirmed dengue infection and different clinical diagnoses (Figure 2). Both C3a (Figure 2A) and C5a (Figure 2B) were present at approximately three-fold higher levels in the DHF patients than in the DF patients; these differences were statistically significant (p<0.001). The elevated levels of C3a and C5a that we observed in DHF patients are consistent with their decreased levels of C3 and further confirm the association of high complement activity with DHF.

### The increased complement activation in DHF patients is correlated with MBL oligomerization, but not correlated with levels of immune complexes, C1q or CRP

**Figure 3:**
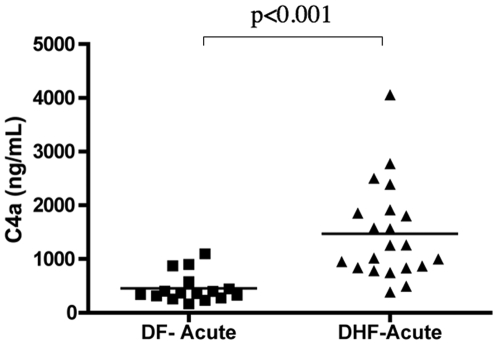
Levels of C4a; plotted values are nonlog for ease of comprehension. Plasma levels of C4a in dengue-infected individuals during the acute phase and in healthy subjects. C4a levels were measured by cytometric bead assay. Values shown are means and standard deviations. The p-values of two-tailed t-tests on the log-transformed data are indicated above each analysis.

**Figure 4:**
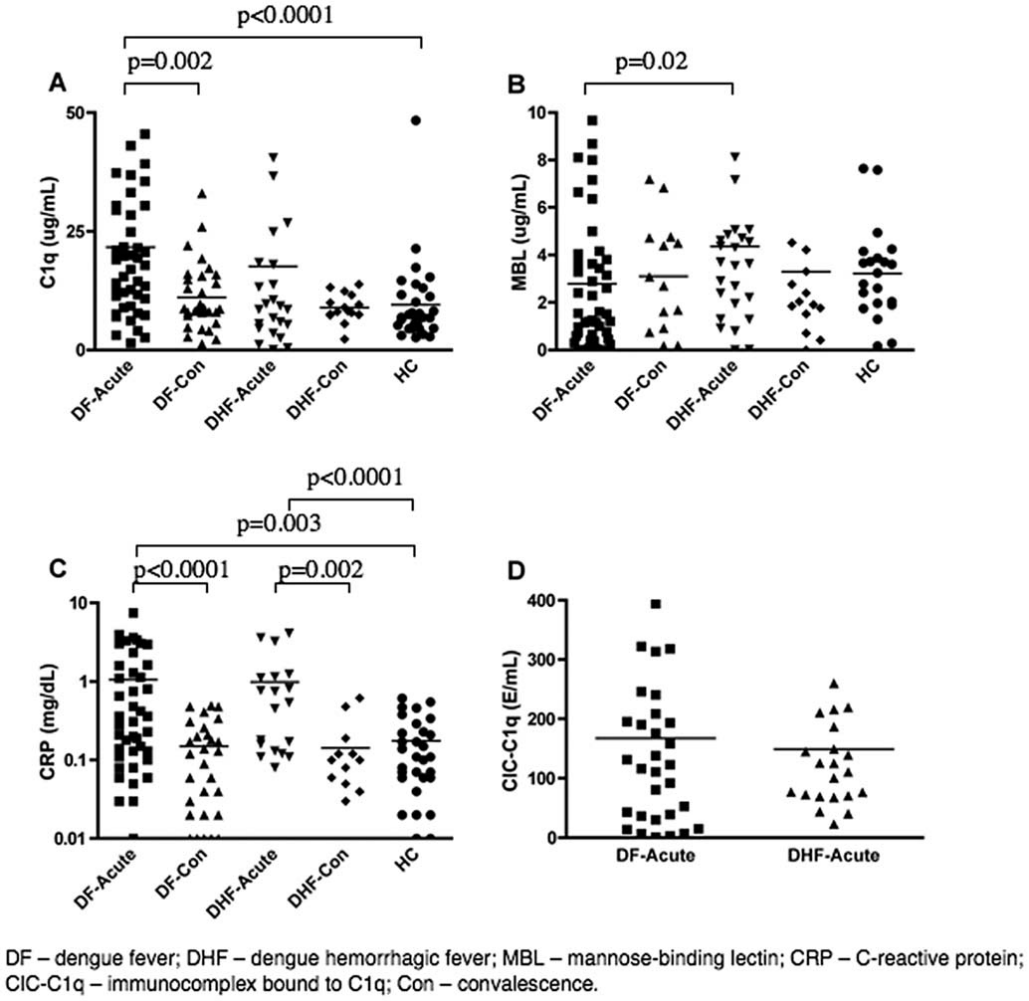
Levels of factors of the CP and LP; plotted values are nonlog for ease of comprehension. (A) Plasma levels of C1q, (B) MBL oligomer, (C) C-reactive protein, and (D) immunocomplex-CIC-C1q in dengue-infected individuals at different stages of infection (acute and convalescent) and in healthy subjects. All complement proteins were measured by ELISA. Values shown are means and standard deviations. The p-values of two-tailed t-tests on the log-transformed data are indicated above each analysis.

The increased consumption of C3 and elevated levels of byproducts of complement activation (C3a and C5a) that we observed in DHF patients confirmed that higher complement activation is correlated with dengue severity. However, the pathway of complement activation was not defined. Therefore, we measured the levels of C4a, which correlates with the formation of the (C4b2b) C3 convertase, a hallmark for activation of the CP or LP (Figure 3). This result indicates that C4a was 3-fold elevated in DHF patients suggesting the involvement of either the CP or the LP. In order to identify the mechanism of the CP or LP activation we measured levels of C1q, CRP, CIC-C1q and oligomerized MBL in plasma samples from DF and DHF patients (Figure 4; Table 2). The levels of C1q and CRP in the DF and DHF patients were elevated during acute infection when compared to healthy control subjects, and they returned to normal levels during convalescence. However, levels of both proteins were similar between DF and DHF patients. There was also no difference in the total IgG immunocomplex levels (CIC-C1q) during the acute phase between the DF and DHF patients. This result suggests that the activation of the (C4b2b) C3 convertase was not mediated by differences on the levels of immune complexes in DF and DHF patients. However, other factors, such as significant differences in the IgG isotype distribution in the two groups could interfere with the interpretation of this result, since different immunoglobulins have different capacities to activate the CP.

The activity of the LP was evaluated by measuring the levels of oligomerized MBL, and a significant increase of MBL oligomerization was found in the DHF patients as compared to the DF patients during the acute phase of infection, which might have contributed to the elevated C4a level. These observations suggest that it is possible that MBL can be contributing to the increased activation of (C4b2b) C3 convertase in DHF through mechanisms that do not involve immune complexes.

### Higher complement activation in DHF patients is associated with abnormal levels of regulatory AP factors

**Figure 5:**
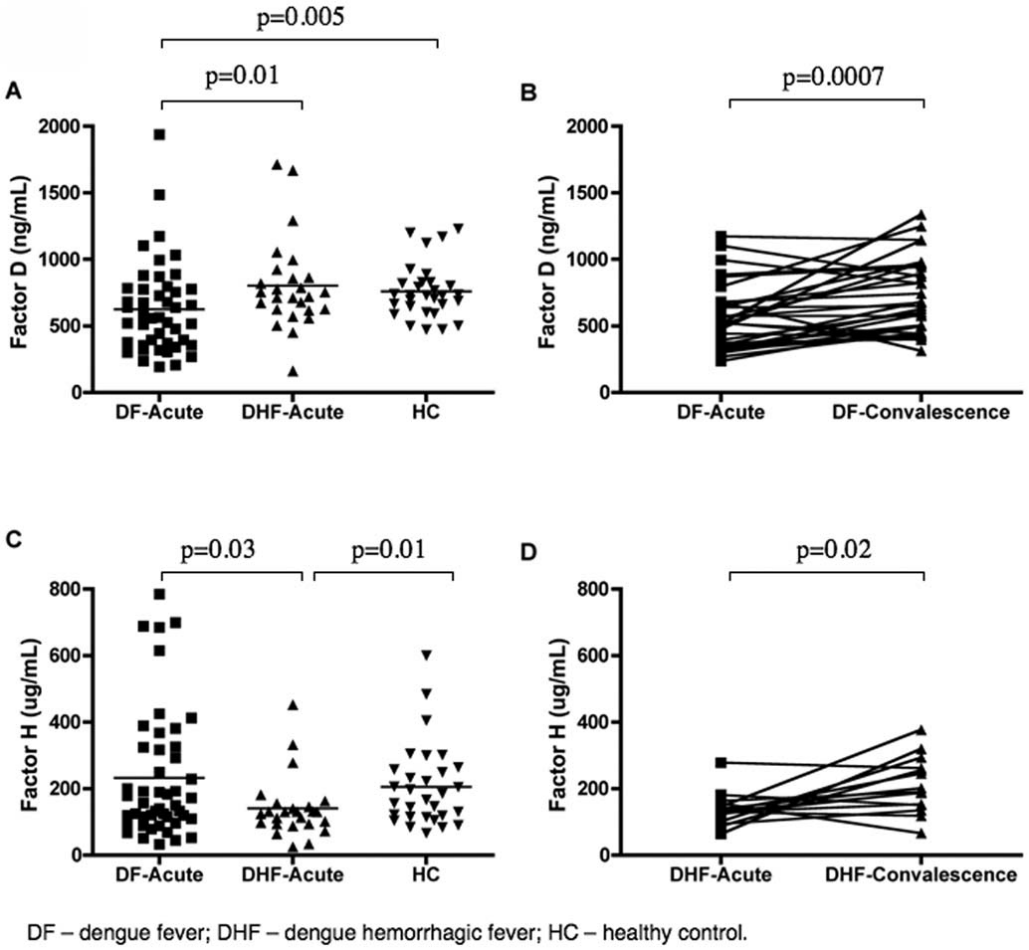
Levels of AP factors; plotted values are nonlog for ease of comprehension. (A) Plasma levels of factor D and (B) factor H in dengue-infected individuals and healthy subjects. (C) Paired comparisons of the factor D levels during the acute and convalescent phases in DHF patients. The mean levels of factor D on DF patients was 555+246 and 704+278 during acute and convalescence phases respectively (D) Paired comparisons of factor H levels during the acute and convalescent phases in DF patients. The mean levels of factor H on DHF patients was 138+49 and 210+83 during acute and convalescence phases respectively All complement proteins were measured by ELISA. Values shown are means and standard deviations. The p-values of two-tailed t-tests on the log-transformed data are indicated above each analysis

Quantitation of two AP components, factors D and H, in DF, DHF, and control patients (Figure 5, Table 2) revealed that the levels of factor D were significantly (p = 0.01) lower in DF patients than in DHF patients during acute infection and also lower than healthy controls (p = 0.005, Figure 5A). During the convalescent phase, the mean factor D levels in the DF patients returned to that of DHF patients and control subjects (Figure 5B), suggesting that the levels of factor D in DF patients were reduced during acute dengue infection, limiting the rate of conversion of the C3bBB into the active C3 convertase, C3bBb, in these patients.

In contrast, the levels of factor H, which displaces Bb from C3bBb and inactivates the C3 convertase, were significantly lower (p = 0.03) in DHF patients than in DF patients during acute infection (Figure 5C). The levels of the factor H in DHF patients were also significantly lower (p = 0.01) than those in healthy individuals, whereas in DF patients, this was not observed. During the convalescent phase, the levels of factor H in the DHF patients had returned to the levels seen in healthy controls (Figure 5D). These data indicate a reduced ability to inhibit the amount of C3bBb C3 convertase in DHF patients. Taken together, the data regarding factors D and H suggest that DHF patients have a reduced ability to regulate the activity of the AP.

**Figure 6:**
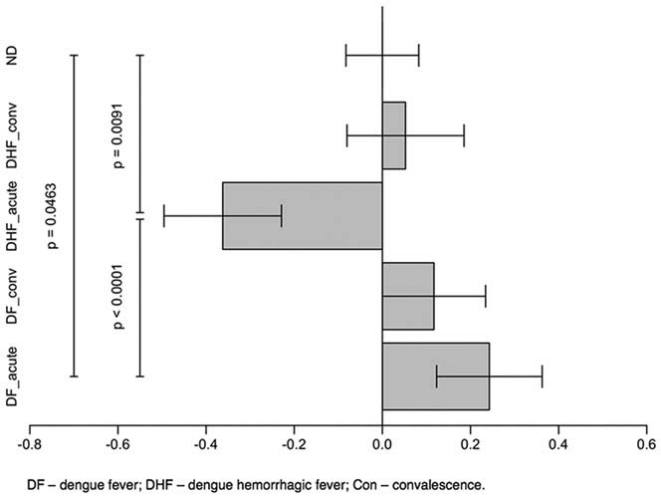
Mean log-ratios of concentration of factor H over the concentration of factor D for each diagnostic category, subtracted from the mean log-ratio in the healthy (ND) category. A value of zero indicates that the levels of both factors were present in the same equilibrium as in healthy control subjects. Negative values indicate reduced AP regulation and favorable formation of (C3aBb) C3 convertase, whereas positive values indicate the opposite. The standard deviation bars give the interval *(mean + err, mean –err)*, where *err* is the usual standard error of difference between sample means, except in the baseline (ND) category, where *err* is the standard error of the sample mean for that category.

**Figure 7:**
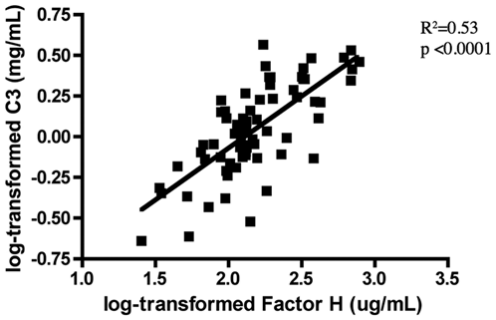
Correlation between levels of factor H and C3. Analysis of correlation was carried out between log-transformed levels of factor H and C3 dengue-infected patients during the acute phase.

Based on the fact that factors D and H act together to influence the availability of the C3 convertase and consequently the activation of the complement system through this pathway, we analyzed the levels of both proteins in each patient through the log-ratio of their concentrations, as described in the Materials and Methods. The results of this analysis indicated that DF and DHF patients exhibited strikingly different patterns of AP activity during acute dengue infection (Figure 6). In this figure, a value of zero indicates that the levels of both factors were at proportions similar to that seen in healthy (ND) control subjects, negative values indicate a greater proportion of factor D in relation to factor H, and positive values indicate the opposite. The acute DF patients showed significantly (p = 0.046) larger log-ratios in relation to healthy controls, whereas the acute DHF patients showed significantly (p = 0.009) reduced log-ratios when compared to healthy controls. The difference in log-ratios between the acute DF with DHF patients was highly significant (p<0.0001). During the convalescent phase, the log-ratios of the DF and DHF patients approximated of those of the control group, and no significant differences were detected (Figure 6). Further supporting the association of the AP regulatory proteins with dengue disease severity, levels of C3 levels and factor H were higly correlated (Figure 7). These results suggest that during acute dengue infection, the regulatory proteins of the AP are modulated in order to maintain the homeostasis of complement activity, and when an imbalance between levels of factor D and factor H occurs, during the acute phase of the infection, the regulation of this pathway is decreased, leading to an abnormal activation of the complement system.

### Increased complement system activity and levels of AP pathway components are correlated with reduced platelet levels

Platelet levels are reduced during acute dengue infection (both DF and DHF), and thrombocytopenia is associated with an increased severity of the dengue infection. The main mechanisms that have been proposed to account for the induction of thrombocytopenia in dengue infection include virus-mediated megakaryocyte suppression in the bone marrow [21] and increased platelet destruction [22]–[24]. In the present analysis, the C3 levels were found to be directly correlated with platelet levels and inversely correlated with C3a and C5a levels (Table 3), indicating that the higher the complement system activity is, the lower the platelet level during acute dengue infection is. Factor H was also highly correlated with platelet levels in the dengue patients, whereas C1q, CIC-C1q, CRP, and MBL protein levels were not (Table 3). These results further support our conclusion that abnormal modulation of AP regulatory proteins is a significant factor contributing to dengue severity.

**Table 3:** Correlations between individual components of the complement system and platelet count in dengue-infected patients during the acute phase.

Protein	Pearson r	95% confidence interval	*p* value	R^2^
**Factor H (ug/mL)**	**0.260**	**0.021 to 0.471**	**0.033**	**0.068**
Factor D (ng/mL)	−0.111	−0.342 to 0.132	0.370	0.012
C1q (ug/mL)	0.194	−0.049 to 0.414	0.117	0.037
MBL (ug/mL)	−0.133	−0.360 to 0.109	0.280	0.018
CIC-C1q (E/mL)	−0.009	−0.283 to 0.267	0.951	0.000
CRP (mg/dL)	−0.022	−0.278 to 0.238	0.872	0.000
**C3 (mg/mL)**	**0.329**	**0.094 to 0.529**	**0.007**	**0.108**
**C3a (ug/mL)**	**−0.525**	**−0.735 to −0.221**	**0.002**	**0.275**
**C5a (ng/mL)**	**−0.494**	**−0.707 to −0.197**	**0.002**	**0.244**
C4a (ng/mL)	−0.1140	−0.4266 to 0.2230	0.5081	0.01299

Proteins in boldface type were significantly (p<0.05) correlated with platelet levels.

## Discussion

The levels of C3, C3a, and C5a that we have measured in dengue patients (Figures 1 and 2) demonstrated the presence of increased complement activity in DHF patients; these findings are consistent with the levels that have been previously reported in patients with this severe form of disease [3], [11], [13], [14]. Our analysis of pathway-specific components of the complement system has suggested that immunocomplexes do not appear to be involved with the abnormal activation of the complement system in DHF patients. However, the complement factors D and H (Figures 5; Table 2), key regulatory proteins of the AP, are present in abnormal levels in DHF patients and an imbalance between the levels of these proteins is correlated with disease severity. The lack of proper regulation of the AP convertase may be responsible for the increased complement activation that has been observed in DHF patients (Figure 6; Table 3).

Factor D, also termed adipsin, is a rate limiting serine protease produced by adipocytes, monocytes, and macrophages [25]. It circulates in active form and is responsible for cleaving BB in the C3bBB complex to produce C3bBb, the active C3 convertase of the AP. Factor D is considered as one of the limiting factors for the activation of the alternative pathway [26]. In our study, levels of factor D in DF patients were lower than those in either DHF patients or healthy controls (Figure 5A), whereas during the convalescent phase, levels of factor D in DF patients were as high as those found in DHF patients during convalescent (Figure 5B). These data suggest that during acute dengue infection, the patients who are more capable of modulating their levels of factor D will exhibit the mild disease phenotype DF. In contrast, it appears that when levels of factor D are not reduced, the patient develops the most severe form of the disease DHF (Table 2).

Although factor D appears to play a role in determining dengue severity, factor H is also widely regarded as one of the most important regulator of the AP. During acute infection, the plasma levels of factor H in DHF patients were lower than those in either DF patients or healthy subjects (Figure 5C), but they returned to normal levels during convalescence (Figure 5D). In contrast, the levels of factor H did not change in the DF patients (Table 2). One possibility is that DHF individuals have a limited capacity to sustain normal factor H levels during acute dengue infection because of genetic factors. Indeed, preliminary data from Acioli-Silva et al. [27] have suggested that individuals with a polymorphism that is associated with increased mRNA expression of factor H have a reduced susceptibility to developing DHF. Factor H gene dysfunctional polymorphisms have been associated with vascular diseases such as age-related retinal degenerative maculopathy and atypical hemolytic uremic syndrome (aHUS) [28]–[30], and the occurrence of these clinical manifestations has also been described in severe dengue cases [31], [32]. In addition, the fact that infants normally produce less factor H than do adults [33] and that this age group is more affected by DHF [34] also supports a possible role for this complement factor in determining dengue severity. Interestingly, studies on NS1 protein from West Nile virus have shown that this protein can bind to factor H, recruiting this complement protein to the surface of infected cells, allowing NS1 bound cells to escape from complement-mediated lyses [15]. However, this mechanism has not been shown to be happening during dengue infection.

On the basis of the evidence obtained so far, especially due to the presence of elevated C4a, we cannot rule out the possibility that CP and/or LP are also involved in determining dengue severity. In the report presented by Bokish et al, the consumption of C4 was mild in DHF patients grades I and II, suggesting that the (C4b2b) is not the main C3 convertase involved in the complement activation in the milder DHF these cases. The (C4b2b) C3 can be activated by several mechanisms including immune complexes, CRP, Specific ICAM-3 Grabbing Non-Integrin (SIGN-1), phosphatidyl serine, complex polysaccharides, and others. Often the CP is triggered by the binding of C1q to either antibody-antigen immunocomplexes [4] or reactive-C protein [5]. Fc-dependent complement-mediated antibody responses are one of the main humoral immune effector mechanisms against flaviviruses, and virus opsonization is a major mechanism of viral clearance that operates via complement receptors [2]. In addition, C1q has been shown to restrict ADE [35], [36], a critical mechanism contributing to DHF immunopathology [37]. Thus, the CP can potentially play an important role in dengue patient outcomes at early stages of infection. However, neither C1q levels neither those components that activate this protein (C-reactive protein and IgG immunocomplexes) were correlated with dengue severity (Figure 3); and these findings are consistent with those of several other studies that found no evidence of abnormal levels of immune complexes and CRP in DHF patients [14], [16], [38]. In addition, our C1q finding is also consistent with the findings reported by Bokisch. In this study the C1q values did not fall significantly in less severe grade I and II DHF cases. Actually, in the example shown in the Bokisch manuscript, the C1q level is above normal at all time points [3]. In a separate study, Sobel used the C1q deviation test and found a correlation of C1q deviation with DHF severity. The C1q deviation test is the measurement of the difference in incorporation of labeled C1q into sheep erythrocytes in relation to a standard sample [39]. As the authors pointed out, the C1q deviation assay is highly sensitive to the presence of DNA, which can be very elevated in the serum of dengue patients. The LP could also potentially play a role, leading to elevated levels of C4a. Previously, we have demonstrated that *MBL2* gene polymorphism is correlated with the risk of thrombocytopenia elicited by dengue virus [40] and in this study we saw a significant correlation between the MBL protein and DHF. There are other known mechanisms to activate (C4b2b) C3 convertase without involving C1q, immune complexes or MBL. Among the interesting possibilities are some of the mechanisms related to clearance of apoptotic bodies [41]. Kuraya and collaborators have reported that ficolins can bind to apoptotic cells and activate the (C4b2b) C3 convertase [42].

Individually, factors D and H showed significant differences during acute dengue infection. But since these two factors have antagonistic effects on the modulation of the activity of the C3bBb C3 convertase, the elevated levels of one could compensate for the levels of the other, we decided to analyze ratios of both factors. The increased log-ratios in concentration between these factors in plasma samples from patients with acute dengue infection clearly pointed to an increase in the regulation of the AP in DF patients and a reduced regulation of the AP in DHF patients when compared to healthy controls. These results suggest the presence of an increased availability of the C3bBb C3 convertase in DHF patients that may be responsible for the higher consumption of C3 and increased production of C3a and C5a in these patients. This observation is consistent with previous direct measures of alternative pathway activaty reported by Bokisch et al. [3].

Our observation that levels of C3 were directly correlated with the platelet levels and inversely correlated with C3a and C5a levels (Table 3) is consistent with previous reports that C3 consumption and C3a and C5a production are correlated with the thrombocytopenia seen in people developing the most severe form of the disease [43], [44]. The complement factors of the CP that we examined here did not show any direct correlation with platelet levels; however, factor H did show strong correlation, supporting our conclusion that the lack of AP regulation is one of the mechanisms contributing to complement activation and disease severity. Interestingly, factor H deficiencies are associated to several diseases involving thrombocytopenia and microvasculopathy [45]–[49]. However, other mechanisms could also contribute to the thrombocytopenia, since some patients have antibodies that cross-react with platelets and endothelial cells and therefore augment the cytotoxicity mediated by the CP [43], [50].

Overall, the majority of the data presented here point to an association between the improper modulation of AP regulatory proteins and increased dengue severity. In addition, previous biochemical evidence supports the existence of an association between dengue proteins and the AP. Whereas membrane-associated NS1 requires the presence of anti-NS1 antibodies to achieve complement activation [11], [51], soluble and purified dengue NS1 protein has been shown to activate complement in the absence of anti-dengue antibodies, suggesting that soluble NS1 can directly induce complement activation [11], [51]. Taken together, these data suggests that AP regulatory proteins such as factor H and factor D, as well as their substrates, are potential therapeutic targets to prevent the onset of DHF.
